# Coupled Finite Element Model of the Middle and Inner Ear as Virtual Test Environment for Stapes Surgery

**DOI:** 10.1002/cnm.70013

**Published:** 2025-02-03

**Authors:** D. Burovikhin, M. Lauxmann

**Affiliations:** ^1^ Reutlingen Research Institute Reutlingen University Reutlingen Germany; ^2^ Reutlingen University Reutlingen Germany

**Keywords:** finite element, inner‐ear model, middle‐ear model, performance measures, stapes prosthesis, virtual test environment

## Abstract

In order to evaluate the performance of different types of middle‐ear prostheses, a model of human ear was developed. The model was created using finite element (FE) method with the ossicles modeled as rigid bodies. First, the middle‐ear FE model was developed and validated using the middle‐ear transfer function measurements available in literature including pathological cases. Then, the inner‐ear FE model was developed and validated using tonotopy, impedance, and relative BM motion level curves from literature. Both models are based on preexisting research with some improvements and were combined into one coupled FE model. The stapes in the coupled FE ear model was replaced with a model of a stapes prosthesis to create a reconstructed ear model that can be used to estimate how different types of stapes protheses perform relative to each other as well as to the natural ear. The influence of the diameter of the prosthesis as well as the influence of the sealing and opening of the gap in the footplate were investigated along with different measures such as maximum basilar membrane displacement, intracochlear pressure, pressure in scala vestibuli, oval and round window volume displacements, and prosthesis displacement. This will help in designing new innovative types of stapes prostheses or any other type of middle‐ear prostheses, as well as to improve the ones that are already available on the market.

## Graphical abstract

In order to evaluate the performance of middle‐ear prostheses, a coupled finite element (FE) model of the human ear was developed. The stapes was replaced with a stapes prosthesis to create a reconstructed ear model. The hearing performance of the reconstructed ear was investigated with different measures such as maximum basilar membrane displacement, intracochlear pressure, pressure in scala vestibuli, oval and round window volume displacements, and prosthesis displacement.

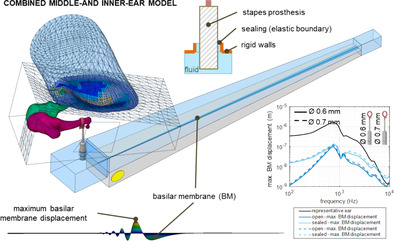

## 1 Introduction

Piston stapes prostheses are commonly used to treat otosclerosis—a medical condition in which the mobility of the stapes is severely hindered by abnormal bone growth in the oval window (OW) and the subsequent calcification of the annular ligament. This leads to a significant loss of conductive and mixed hearing. According to Ahmad et al. [1], otosclerosis occurs most commonly in Caucasians with an incidence of 1%, followed by Asians at 0.5%. Otosclerosis tends to present in the third and fourth decades of life, and presents more commonly in women with a ratio of approximately 2:1. Piston‐stapedotomy is an effective treatment for this condition. During stapedotomy, a small hole is drilled in the calcified stapes footplate (SF), the cylindrical end of the piston prostheses is inserted into the hole and the other end is crimped around the long crus of the incus. The prosthesis stimulates the inner‐ear fluid and partially restores the lost hearing. A typical stapes prosthesis consists of a cylindrical piston at one end with the diameter varying between 0.4 and 0.8 mm and a wire with different attachments at the other end.

Experiments with cadaveric temporal bones are commonly performed to assess the hearing performance of reconstructed ears using the stapes volume displacement as an evaluation factor representing the effective stimuli to the cochlea. In this study, other measures such as the piston velocity of the stapes prosthesis, the basilar membrane (BM) displacement, and the intracochlear pressure difference are investigated and compared to each other and to the net volume flow in the cochlea. As a reference, we use measurements from the literature. Sim et al. [2] measured the net volume flow in the cochlea at the round window (RW) in order to assess objectively the outcome of a stapedotomy surgery. Raufer et al. [3] determined the middle‐ear actuator performance from intracochlear pressure measurements in a single cochlear scala. However, temporal bone measurements often have several limitations:

- Executing the measurements often requires a considerable amount of time, particularly due to the lengthy process of obtaining approval from the ethics council.
- They show significant variations in the results due to the variation across subjects.
- Exact and reproducible results are very difficult to achieve. This makes it difficult to evaluate different designs of a prosthesis during the development process.
- The number of samples that can be tested is limited (5–20 samples per study).

As a result, the product optimization cycles stretch over several months, which substantially slows down the development process. Instead, an FE model can be used to evaluate the surgical performance of prostheses.

There is a number of FE models of the inner and middle ear developed for various purposes. A good overview can be found in Paolis et al. [4]. The FE model presented in this study is based on preexisting research. The middle‐ear model was adopted from Sackmann et al. [5] where its parameters were optimized not only for a healthy ear, but also for a number of common pathologies. The inner‐ear model was based on the models from Gan and Wang [6] and Kwacz et al. [7], but some of the geometry and material properties were changed to achieve a better fit to measurements from literature. Not only the inner‐ear model was validated with regard to tonotopy and relative BM motion level measurements, but also the inner‐ear impedance measurements and net volume flow in the cochlear were taken into consideration. The FE model of the inner ear presented in this study is a straight two‐channel model. There are other FE models where cochlea is modeled as a coiled body such as Areias et al. [8], Zhang et al. [9], and Zhang and Gan [10], which aim to provide a more anatomically accurate model of the human ear, however, these more complex models do not provide any additional significant benefits when it comes to the goals of the present study.

The aim of this study is to develop an FE model of the middle ear coupled with the inner ear that can be potentially used to evaluate the performance of different types of middle‐ear prostheses. Special focus is given to stapedotomy and the measures that can be used to evaluate its functional outcome and the performance of the stapes prostheses.

## 2 Material and Methods

### 2.1 Inner‐Ear Model

**FIGURE 1:**
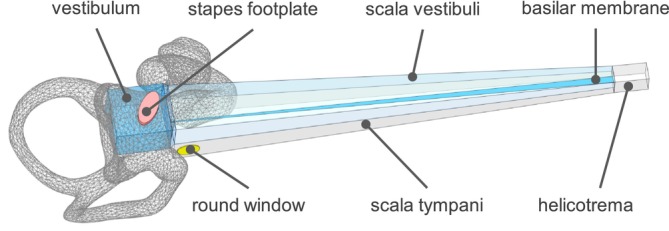
Overview of the inner‐ear model.

The inner and middle ear is modeled using ANSYS 2021 R2. The inner‐ear model consists of OW, vestibulum, scala vestibuli (SV), BM, scala tympani (ST), and RW as shown in Figure 1. A simplified SF is used to couple the middle‐ear to the inner‐ear model.

The vestibulum is modeled as a solid body meshed with 4780 acoustic fluid quadratic elements FLUID220. The volume of the vestibulum is 37.8 mm^3^, which approximately corresponds to the volume of 40 mm^3^ as stated in Buckingham and Valvassori [11]. Scanned CT geometry of the cochlea is used as a reference to realistically model the location of the OW and RW and the location of the entrance to the SV, as well as the orientation and the shape of the vestibulum as shown in Figure 1.

**FIGURE 2:**
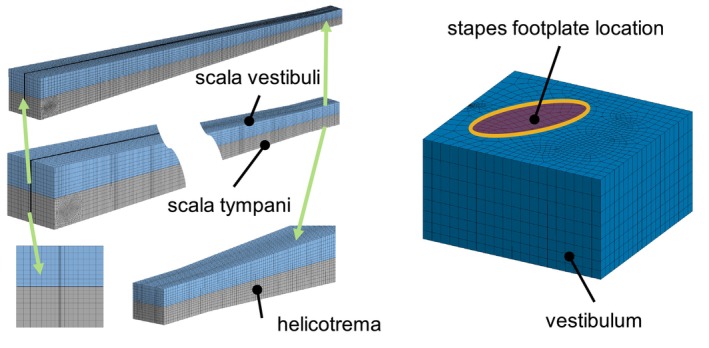
Mesh of the scalae and vestibulum.

The spiral canal of the cochlea is modeled as an uncoiled two‐channel system meshed with 33,916 acoustic fluid quadratic elements FLUID220 with compressible formulation, as shown in Figure 2. This assumption is accepted in general for the study of cochlear mechanics using mathematical, FE, and physical models [6]. In the cochlear model, the effect of scala media chamber is not considered and the micromechanical structure of the organ of Corti is not included. The dimensions of the cochlear chambers as well as of the OW, RW, SV, ST, and BM are based on the values published in Wahl [12], Kwacz et al. [7], and Gan and Wang [6], but some modifications were made to improve the model. This includes the introduction of the vestibulum volume that is not present in other models, the adjustment of the dimensions and positions of the SF and RW according to the data from literature and the adjustment of the dimensions and the position of the cochlear chambers which was done to get a better fit to the published measurements.

**FIGURE 3:**
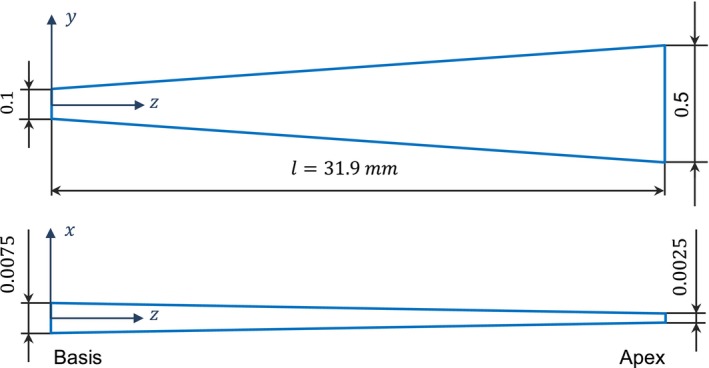
Basilar membrane geometry given in millimeter.

The BM separates the scalae and is modeled as a thin volume meshed with 4850 solid quadratic elements SOLID186 with and elements along the thickness of the membrane. The geometry of the BM is shown in Figure 3. Both the thickness and the width vary along the length of the membrane.

It is established in Gan and Wang [6] that the Young's modulus and damping properties of the BM are inhomogeneously distributed along its length. In this study, the BM is modeled using an isotropic material law. The Young's modulus value varies continuously along the length of the membrane on element‐by‐element basis, which reflects a gradual change of the volume fraction of the fibers along the length of the membrane. The values themselves are interpolated using a quadratic polynomial, where x is the distance from the base of the BM.

(1) $$E=-28732\cdot x^{2}-557\cdot x+50\;\left(\mathrm{MPa}\right),0<x\leq 31.9\cdot 10^{-3}\;m$$

The damping properties of the BM are modeled using beta damping (Rayleigh damping) with a linearly changing beta damping factor $\beta_{1}$ from 0 to 20 mm and a quadratically changing beta factor $\beta_{2}$ from 20 to 31.9 mm.

(2) $$\begin{array}{c} \beta_{1}=45\cdot 10^{-5}\cdot x+10^{-6}\;\left(s\right),\;0<x\leq 20\cdot 10^{-3}\;m\\ \beta_{2}=3.43\cdot x^{2}+0.13\cdot x+0.0012\;\left(s\right),\;20\cdot 10^{-3}<x\leq 31.9\cdot 10^{-3}\;m \end{array}$$

**FIGURE 4:**
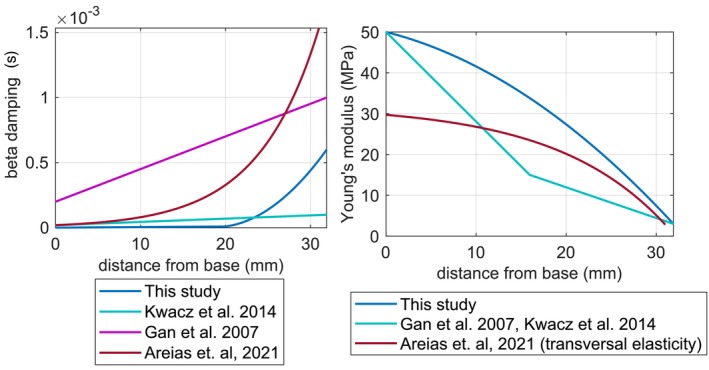
Basilar membrane material and damping properties.

Equations (1) and (2) are a result of a fitting process where the simulated tonotopy curve was matched with the measured tonotopy curve defined in Greenwood [13], which is described by Equation (3) in the results section. Although damping is primarily caused by the viscous effects of the fluid, we chose to model the fluid as inviscid to simplify certain simulation aspects such as meshing, simulation time, and fluid–structure coupling. Therefore, our material model of the BM does not reflect the actual material properties of the BM, but rather has a descriptive character that encapsulates the BM and its adjacent structures. The values of Young's modulus and beta damping are calculated with the help of APDL commands for each finite element of the BM based on its proximity x to the base. A similar approach of defining the Young's modulus and beta damping values is used in Areias et al. [8], where in contrast to this study, the BM was modeled as a spiral using an orthotropic material law under plane‐stress conditions, whereby the longitudinal elasticity modulus was 10 times lower than the transversal. Figure 4 compares the different models from Kwacz et al. [7], Gan and Wang [6], and Areias et al. [8].

**FIGURE 5:**
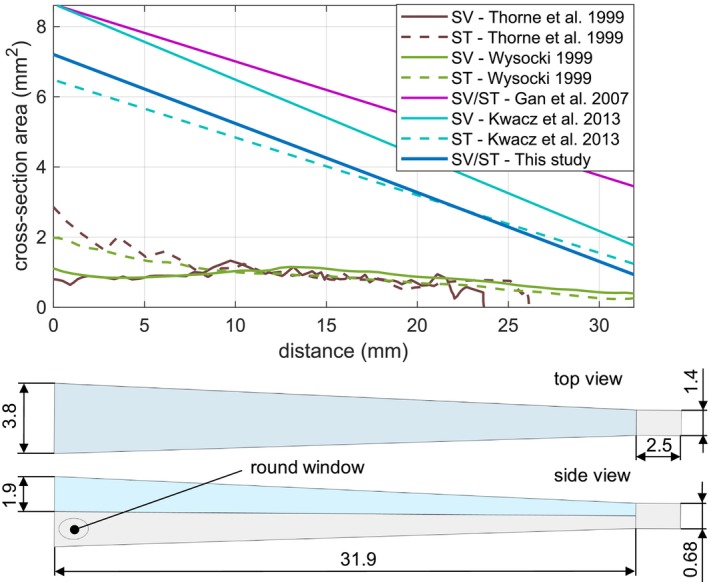
Scalae cross‐section area and geometry given in millimeter. The anatomical measured values are taken from the papers of Thorne et al. and Wysocki et al.

Both SV and ST have the same geometry. At the apex region, the SV and ST are connected through a small opening called the helicotrema. Figure 5 shows the geometry of the scalae in the top view and the view from the side, in which the RW is visible and compares the cross‐section area to the anatomical measurements from Wysocki [14] and Thorne et al. [15] and model values from Kwacz et al. [7] and Gan and Wang [6]. The cross‐sections of the model are of similar order of magnitude, however, they are significantly greater compared to the anatomical cross‐sections. This is due to the fact that in two‐channel models, the fluid volume of the scala media is assigned to the other two scalae. Furthermore, without the damping effect of the organ of Corti, tectorial membrane, and hair cells, a higher mass loading is necessary, resulting in larger cross‐sectional areas required to match the cochlear input impedance in the models to the measured values.

**FIGURE 6:**
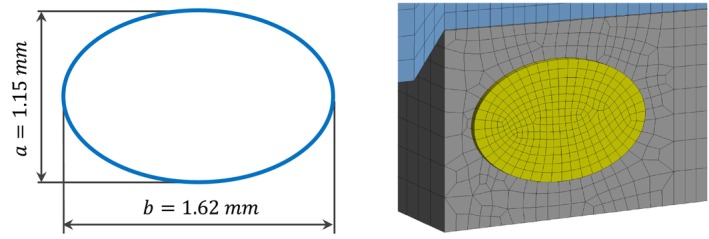
Round window—dimensions and mesh.

According to Singla A. [16], the average height and width of the RW is 1.62 ± 0.77 mm and 1.15 ± 0.39 mm, respectively, as shown in Figure 6. The RW is meshed with solid quadratic elements SOLID186 with three elements along the thickness. The thickness of 0.06 mm is chosen according to Heckeler [17].

**FIGURE 7:**
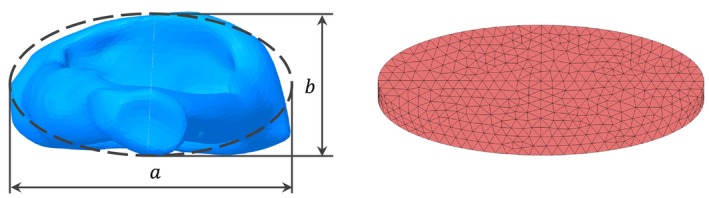
Stapes footplate—dimensions and mesh.

The SF is modeled as an elliptical volume with the area of approximately 2.8 mm^2^. The footplate area is chosen according to Sim et al. [18], where it was determined that the length of the footplate *a* is 2.81 ± 0.158 mm and the width *b* is 1.27 ± 0.109 mm. The footplate volume is meshed with solid quadratic tetrahedral elements SOLID187 as shown in Figure 7.

All the surfaces of the fluid bodies have a rigid wall boundary condition applied to them, unless a fluid structure interface is defined between the faces of the fluid and solid bodies. In this model, the contact regions between the vestibulum and the footplate, between the RW and ST, between the ST and the BM, and between the SV and the BM have a fluid structure interface and all the other surfaces have the rigid wall boundary condition applied to them. The material properties of the inner‐ear model are summarized in Table S1. The footplate is massless and is used to apply harmonic loads (velocity or displacement excitation) to the inner‐ear fluid.

### 2.2 Middle‐Ear Model

**FIGURE 8:**
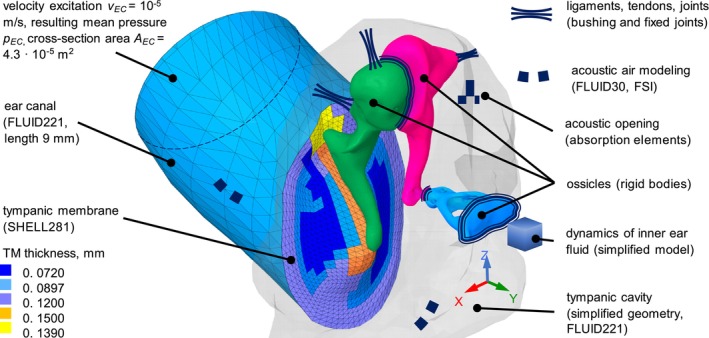
The FE model of the middle ear. The velocity excitation of 10e−5 m/s corresponds to 94 dB SPL.

The FE middle‐ear model used for this study is developed in ANSYS 2021R2 and shown in Figure 8. The model is based on the model developed by Sackmann et al. [5] and Sackmann et al. [19], which is created in Hypermesh (Altair Engineering Inc.). The geometry of the TM, the ossicles, the ear canal, and the tympanic cavity is reconstructed using micro‐CT.

The TM is divided into five regions with a constant thickness at each region, which are derived from the characteristic relative thickness distribution measured in van der Jeught et al. [20]. The five thickness regions are represented by five different colors. The TM is meshed using second‐order shell elements Shell181. All shell elements have the same structural damping coefficient. Only the translational degrees of freedom (DOF) of the TM's outer edge are fixed.

The ear canal and tympanic cavity is meshed with second‐order Fluid221 tetrahedral elements. The aditus ad antrum and the air in the mastoid are modeled as a one‐mass oscillator, which represents the oscillating air between the tympanic cavity and the mastoid, and corresponds to a Helmholtz resonator. The coupling between the TM and the adjacent fluid volumes (EC and TC) is implemented using fluid–structure interaction (FSI) interface in ANSYS Mechanical. To define the FSI, a bonded contact is used between the structural and fluid nodes. That means there is a no‐slip condition between the nodes of the fluid and the structure between which the FSI is defined. The forces at the structural nodes are proportional to the pressure of the fluid in contact with the structural nodes. On the other hand, the pressure on the fluid nodes is proportional to the nodal inertia load of the structural nodes in contact with the fluid nodes which is in turn proportional to the nodal accelerations. The ear canal and tympanic cavity walls are considered rigid which is a default boundary condition for acoustic bodies in ANSYS Mechanical. The geometry of the tympanic cavity is simplified by using plane side walls. According to Whittemore K.R. et al. [21] and Gyo K. [22], the volume of the tympanic cavity varies from about 0.51–0.85 mL. In this study, the volume of the cavity is 0.63 mL. A boundary condition with acoustic absorption elements was used to model acoustic openings of about 2–3 mm in diameter in the tympanic cavity due to the facial recess access in the referenced temporal bone experiments [5].

A modal analysis for each unconstrained ossicle was conducted in ANSYS. The following material properties are assumed: Young's modulus 1.2e10 N/m^2^, Poisson's ratio 0.3, and a homogenized density of the compacta and spongiosa bone layers of 2400 kg/m^3^. The resulting eigenfrequency of the first elastic deformation of the ossicles is above 30 kHz (malleus: 29.8 kHz; incus: 31.3 kHz; stapes: 51.5 kHz). This leads to the assumption that up to 30 kHz, the behavior of the ossicles is stiffness‐dominated and their elastic deformation can be safely neglected in the frequency range up to 10 kHz. Hence, the ossicles are modeled as rigid bodies. The malleus and the incus are modeled having all 6 DOF, whereas the stapes is constrained, allowing only a translational piston‐like motion along the lateral–medial *y*‐axis and two rotational (rocking‐like) motions around the anterior–posterior *x*‐ and superior–inferior *z*‐axis of the stapes coordinate system.

The ligaments, tendons, and joints are modeled as bushing joints with multiple point constraint (MPC) formulation. For the anterior mallear ligament and posterior incudal ligament, stiffness and damping properties are nonzero for all 6 DOF. The superior and lateral mallear ligament as well as the tensor tympani tendon have only one nonzero translational stiffness and damping parameter along their main geometrical axis. To further reduce the number of parameters, the stapedial tendon is removed from the model. The annular ligament model is simplified to only three decoupled stiffness and damping properties, corresponding to the 3 DOF of the stapes. In this middle‐ear FE model, the dynamics of the inner ear are simplified to an overdamped one‐mass oscillator. This simplified inner‐ear model is replaced by a comprehended inner‐ear model described in the present study.

In the harmonic analysis, the air in the ear canal is excited with a frequency independent velocity $v_{\mathrm{EC}}$ of 10^−5^ m/s, which is uniformly applied over the cross‐section at the inlet of the 9 mm long, shortened ear canal, and therefore corresponds to an ideal sound source.

### 2.3 Coupled Model

**FIGURE 9:**
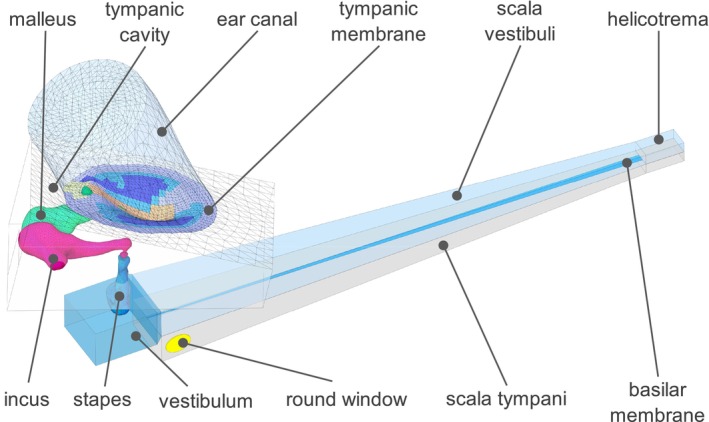
Combined middle‐ and inner‐ear model.

The combined ear model is shown in Figure 9.

**FIGURE 10:**
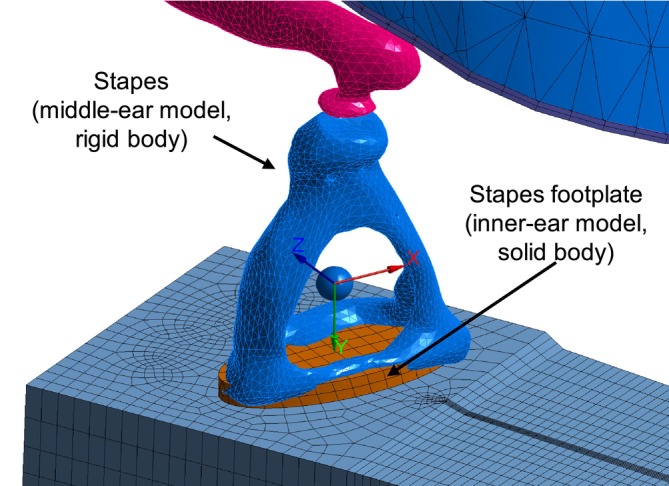
Connection between the inner‐ear and middle‐ear models.

The two models are coupled by rigidly connecting the stapes of the middle‐ear model, which is a rigid body, to the footplate of the inner‐ear model represented by a massless and stiff solid body as shown in Figure 10. This connection is modeled using a fixed joint.

### 2.4 Coupled Model With Stapes Prosthesis

**FIGURE 11:**
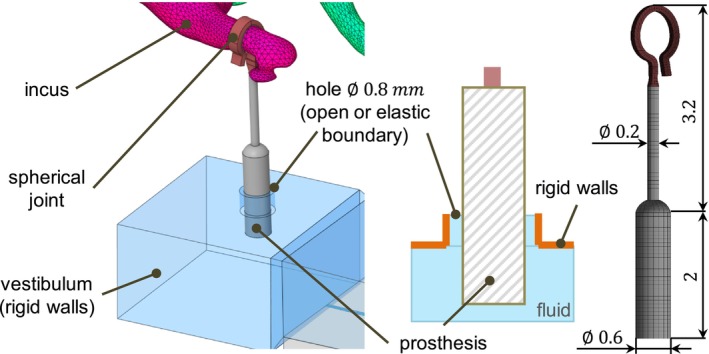
An overview of the combined ear model with the stapes prosthesis.

The stapes is replaced with a stapes prosthesis consisting of a cylinder with the diameter of 0.6 mm and the length of 2 mm and a wire with the diameter of 0.2 mm (Figure 11). The connection between the hook of the wire and the incus is idealized by modeling it as a spherical joint, whose reference coordinate system lies at the center of the prosthesis loop. This means that the relative rotations around the center of the joint are free and the relative translations are fixed. It is assumed that the hook does not typically lie flush with the surface of the lenticularis and is in a line contact rather than a surface contact. This leads to the assumption that the connection has a high stiffness in translational directions and a very low rotational stiffness. Furthermore, this modeling approach neglects postoperative tissue formation between the incus and the hook. The SF is replaced by the rigid wall boundary condition as shown in Figure 11. The diameter of the hole through which the prosthesis is inserted into the inner ear is 0.8 mm.

The fluid in the hole is modeled as an extension of the vestibulum volume. The gap is modeled as open and sealed, respectively, as shown in Figure 11. In the case of the sealed gap, an elastic wall boundary with the properties of a soft tissue like the tympanic membrane is used—the Young's modulus is assumed to be 20 MPa. The outer walls of the fluid volumes have a rigid wall boundary condition.

Due to complex geometry, the vestibulum is re‐meshed with 7896 quadratic fluid tetra elements FLUID221. The piston of the prosthesis is meshed with 977 quadratic solid tetra elements SOLID187. The wire of the prosthesis is meshed with 1485 quadratic solid hex elements SOLID186. The simplified SF is mesh with 390 quadratic solid tetra elements SOLID187. All the contact faces between solid and fluid bodies have fluid structure interface. To achieve a consistent mesh at the contact faces, shared topology feature is used for the piston and the vestibulum. This means that nodes of the fluid structures at the interface to the prosthesis are coincident and shared with nodes of the prothesis.

**FIGURE 12:**
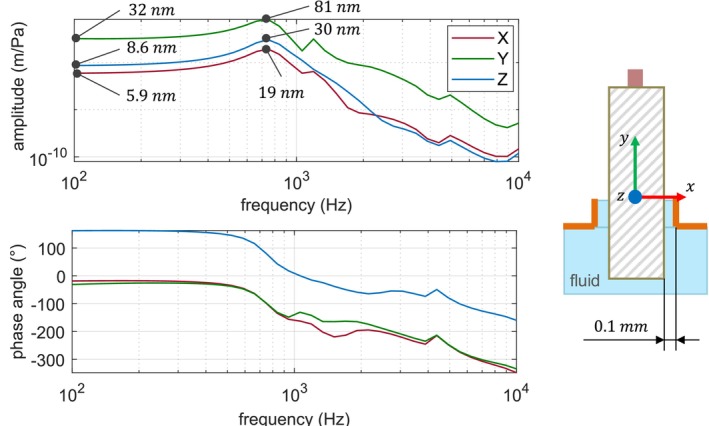
Spatial motion of the prosthesis in the footplate plane during acoustic sound transfer (94 dB SPL).

The piston is naturally constrained in the footplate plane (i.e., in the *xz*‐plane) by the inertia of the fluid. No additional constraints are necessary as our simulations show that the lateral movement within the footplate plane remains below 0.1 mm during acoustic sound transfer, corresponding to the width of the gap between the prosthesis and the footplate illustrated in Figure 12.

## 3 Results

### 3.1 Middle‐Ear Transfer Function

**FIGURE 13:**
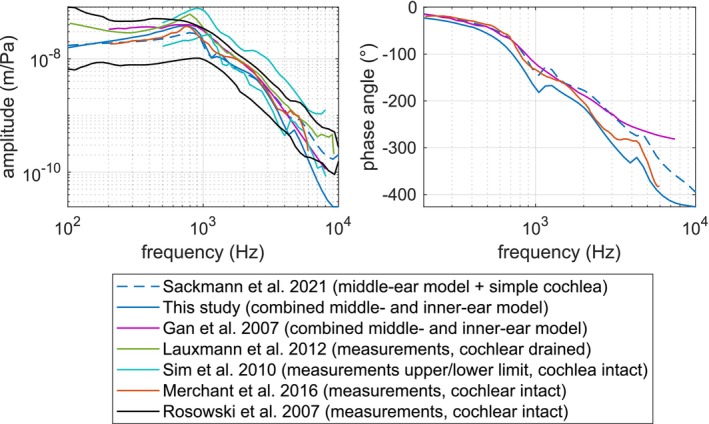
Middle‐ear transfer function: stapes footplate center motion in the *y*‐direction referred to the pressure at the EC inlet.

The simulated middle‐ear transfer function (METF) with the simplified and more extensive inner‐ear models is shown in Figure 13. The METF shows the frequency response of the stapes displacement measured at the footplate center in the *y*‐direction (normally denoted as the stapes piston‐like motion) and referred to the pressure in the ear canal measured at its inlet, which in this study is located at the distance of 9 mm from the tympanic membrane. There is a clear difference between the ear models coupled with the simplified and the extensive inner‐ear models, that can be explained by inner‐ear impedance. The difference is more noticeable in the low and high frequencies where the inner‐ear impedance has its highest values as seen in Figure 16. Despite that, the METF still fits well within the boundaries derived from the measurements in Merchant et al. [23] and Sim et al. [24]. Going forward, the combined model is used as the representative ear which serves as a reference when evaluating the performance of the stapes prostheses.

### 3.2 Tonotopy Curve

**FIGURE 14:**
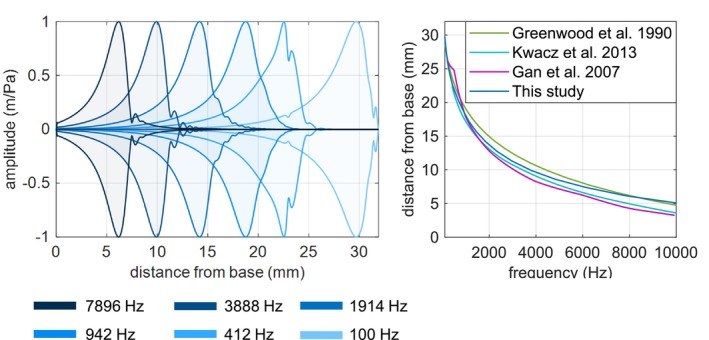
Left: vibrational response of the basilar membrane (94 dB SPL). Right: distribution of the characteristic frequency of the BM.

Tonotopy curve represents the distribution of the characteristic frequency along the length of the BM. In Figure 14, the deformation amplitudes of the BM are shown. The location of the maximum amplitude depends on the excitation frequency. It can be seen that low frequencies primarily excite the BM at the apex, whereas higher frequencies excite it more at the basis. The tonotopy curve shows how the location of the amplitude of the BM peak displacement depends on the excitation frequency. Greenwood has measured this dependency in Greenwood [13] and derived a formula that describes it mathematically for a passive cochlea:

(3) $$F=A\cdot \left(10^{\alpha \cdot \left(1-\frac{x}{31.9}\right)}-k\right)$$

where suitable constants (for men) are: *A* = 165 to yield characteristic frequency *F* in Hz at the distance *x*, *α* = 2.1, if *x* is expressed as a proportion of the BM length. The latter constant is an empirical constant arising in the critical‐band function. The integration constant *k* was left at the value of 1, but it may sometimes be better to replace it by a number in the range from 0.8 to 0.9, to set a lower frequency limit dictated by convention or by the best fit to data. Thus, the value *k* = 0.88 would yield the conventional lower frequency limit of 20 Hz for men [13]. Variable *x* is the distance from the base of the BM and it varies from 0 to 31.9 mm which is the length of the BM. Figure 14 also shows how our simulation model compares to the inner‐ear models developed by Kwacz et al. [7] and Gan and Wang [6].

### 3.3 Relative Basilar Membrane Motion Level

The relative BM motion level is defined as the BM displacement related to the SF displacement at 12 mm distance from the base. It is expressed in dB and calculated as:

(4) $$\text{Relative}\;\mathrm{BM}\;\text{motion level}=20\cdot \log_{10}\frac{{\text{disp}}_{\mathrm{BM}\;\mathrm{at}\;12\;\mathrm{mm}}}{{\text{disp}}_{\mathrm{SF}}}\;\left(\mathrm{dB}\right)$$

**FIGURE 15:**
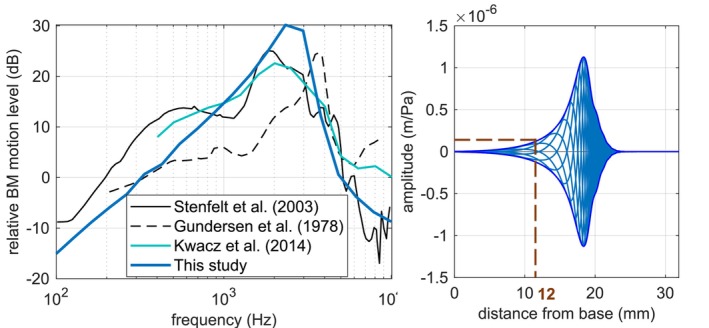
Left: relative BM motion level at 12 mm from the base. Right: vibrational response of the BM at 1833 Hz.

The simulation results are compared to the measurement results obtained by Stenfelt et al. [25] and Gundersen et al. [26] and simulated results from Kwacz et al. [27]. The comparison is shown in Figure 15.

### 3.4 Inner‐Ear Impedance

Inner‐ear (input) impedance is defined as:

(5) $$Z_{\text{inner‐ear}}=\frac{p\mathrm{SV}}{A_{\mathrm{SF}}\cdot V_{\mathrm{SF}}}$$

where $p_{\mathrm{SV}}$ is the pressure at the inlet of the SV and $A_{\mathrm{SF}}\cdot V_{\mathrm{SF}}$ is the volume velocity, $A_{\mathrm{SF}}$ is the area of the SF and $V_{\mathrm{SF}}$ is the velocity of the SF.

**FIGURE 16:**
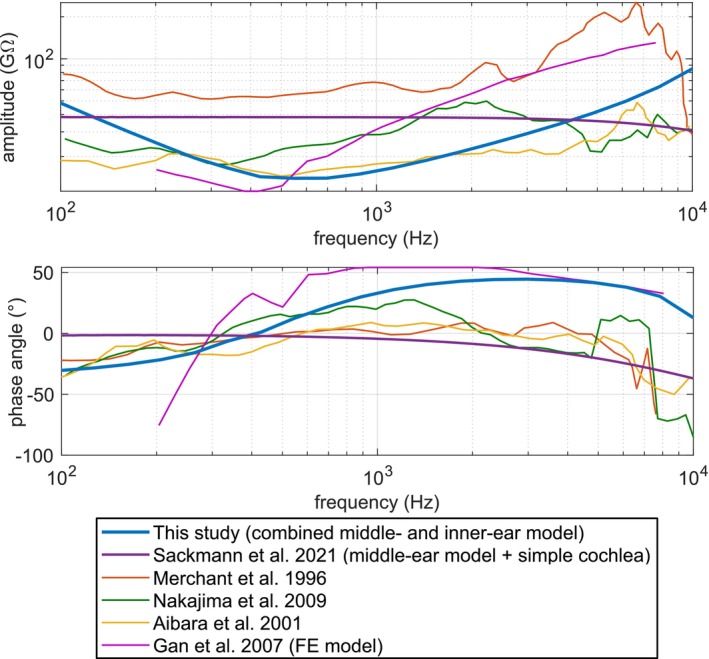
Inner‐ear impedance—measurements versus simulation.

There are several inner‐ear impedance measurements found in literature. The simulation results are compared to the measurements performed by Merchant et al. [28], Aibara et al. [29], and Nakajima et al. [30], and simulated results from Gan and Wang [6]. The comparison is shown in Figure 16.

### 3.5 Stapes Footplate and Round Window Volume Displacement

**FIGURE 17:**
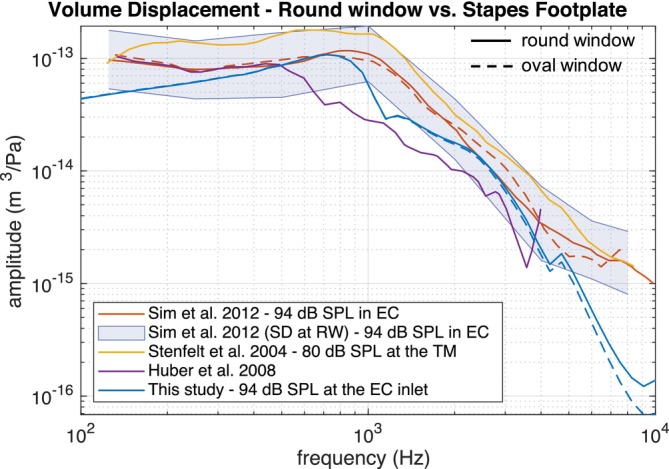
Netto volume displacements at the oval and round windows in normal ears—measurements versus simulation.

In Sim et al. [2], volume displacements at the OW and RW in normal ears were measured on six temporal bones and it was observed that they have roughly the same magnitudes. Our FE model produces similar magnitudes of OW and RW volume displacements as shown in Figure 17, where the simulated results are plotted against the measurements from literature [2, 31, 32]. The stimulation level is 94 dB SPL. The pressure was applied to the ear canal inlet at the distance of about 9 mm from the umbo. The volume displacement is calculated as the product of stapes piston‐like displacement and footplate area defined as π·a·b, where *a* and *b* are the width and the length of the footplate as shown in Figure 7.

### 3.6 Stapes Prosthesis

To estimate the performance of a stapes prosthesis, different measures are commonly used. Besides the maximum BM displacement, there is the pressure $p_{\mathrm{sv}}$ in the SV measured at the OW or the pressure $p_{\mathrm{ST}}$ in the ST measured at the RW or the difference between them, which is denoted as inter‐cochlear pressure difference (ICP). Lastly, there is the volume displacement at the RW VD_RW_. The ratio between the measures in the reconstructed and the representative, natural case, can be considered as an index for conductive hearing loss and thus a kind of index for expected air–bone gap (ABG), assuming that bone conduction is normal:

(6) $$\mathrm{ABG}=20\;\log_{10}\frac{{\text{measure}}_{\text{reconstructed}}}{{\text{measure}}_{\text{representative}}}\;\left(\mathrm{dB}\right)$$

where ${\text{measure}}_{\text{representative}}$ is the measure of corresponding quantity in normal ear, and ${\text{measure}}_{\text{reconstructed}}$ is the measure of corresponding quantity in reconstructed ear.

**FIGURE 18:**
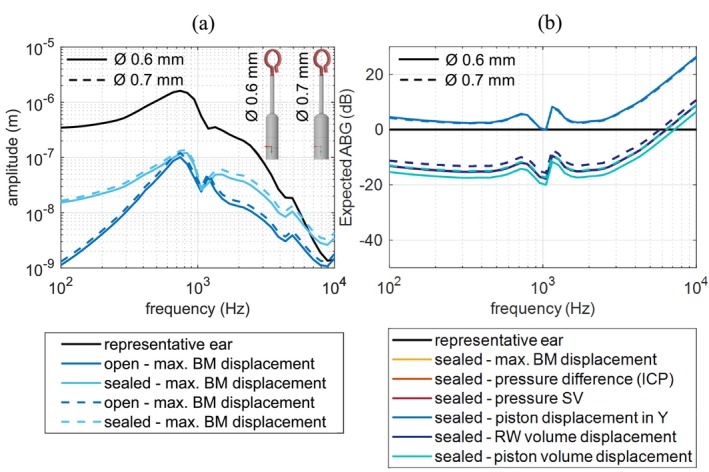
(a) Maximum basilar membrane displacement for the reconstructed (piston *Ø* 0.6/0.7 mm) and representative ear. (b) The comparison of expected ABG calculated in Equation (6) derived for different measures in the case of a sealed gap.

Figure 18a shows, as an example, the absolute values for maximum BM displacement for two stapes prostheses with different piston diameter 0.6 and 0.7 mm differentiating between open and sealed boundary conditions at the gap. Figure 18b shows all the measures mentioned above referred to the representative ear (ABG), in the case where the gap is sealed. Since the maximum BM displacement, pressure difference, pressure in SV, and RW volume displacement curves overlap with each other, only the RW volume displacement curve can be seen in Figure 18. It can be seen that any of the measures except for the piston‐like displacement in *y*‐direction can be used to estimate the performance of the prosthesis, which is very helpful when it comes to evaluating the prostheses performance at different stages of its development and comparing the simulated results to the measurements. According to our simulation, even the pressure measured only in the SV $p_{\mathrm{SV}}$ is enough to evaluate prosthesis performance which corresponds to the findings presented in Raufer et al. [3]. However, the piston‐like displacement (displacement in *y*‐direction) is not affected by the change of the piston diameter and thus it is not a proper measure that could be used to evaluate the performance. This can be explained by the fact that the mass of the prosthesis is much lower than the mass of the ossicular chain. This leads to the prosthesis being displacement driven by the ossicular chain and the slight increase of its inertia in case of a larger piston diameter does not significantly affect its displacement. Overall, according to the simulation, the increase in diameter from 0.6 to 0.7 mm results in the gain of about 1 dB.

By comparing the open and sealed boundary conditions at the gap, it can be seen that there is a significant hearing loss in the lower frequency range before the main resonance at about 750 Hz as well as an approximate loss of 5 dB above 2 kHz when the gap is left open.

## 4 Discussion

The combined ear model was validated using the measurement curves of the METF, tonotopy, relative BM motion level, inner‐ear impedance, and OW/RW volume displacements. The tonotopy and relative BM motion level curves are mainly defined by the mechanical properties of the BM and fit well to the measurements. Areias et al. [8] investigated the influence of a straight and spiral geometry of the BM and different material laws on the tonotopy. A straight‐isotropic, straight‐transversely isotropic, spiral‐isotropic, and spiral‐transversely isotropic models were compared to the Greenwood function, see Equation (3), by means of the root‐mean square errors of 2.05, 1.70, 2.72, and 2.08 mm. For comparison, our straight‐isotropic BM model has a root‐mean square error of 0.84.

The METF and OW/RW volume displacement curves fit to the measurements well, but due to the inner‐ear impedance, there is a small loss in amplitudes in the lower and upper frequency ranges before and after the main resonance at about 750 Hz. The impedance curve itself, although, it fits to the amplitude ranges from the measurements, does not have the same qualitative characteristic with the highest values located at 100 and 10,000 Hz and the lowest at around 600 Hz. This can be explained by the fact that FLUID220 element does not simulate fluid shear viscosity, so it is impossible to reproduce the exact same characteristic of the impedance curves from the measurements with this element. However, it still can be used for the purposes stated in this paper, namely, the evaluation of the stapes prostheses' performance.

It should be also noticed that in the case of the representative ear simulation, there is a small growing difference between the volume displacements at the RW and OW in the simulation at higher frequencies when the inner‐ear fluid is modeled as compressible (see Figure 17). This can be explained by the influence of the first acoustic resonance of the scalae. In the case when the inner‐ear fluid is modeled as incompressible, there is no such difference between the RW and OW volume displacements. Since the compressible fluid formulation is a more physically accurate way of modeling the inner‐ear fluid and the fluid compressibility has a negligible influence on the BM displacement and cochlear pressure distribution, we decided to use the compressible fluid formulation in our FE model.

**FIGURE 19:**
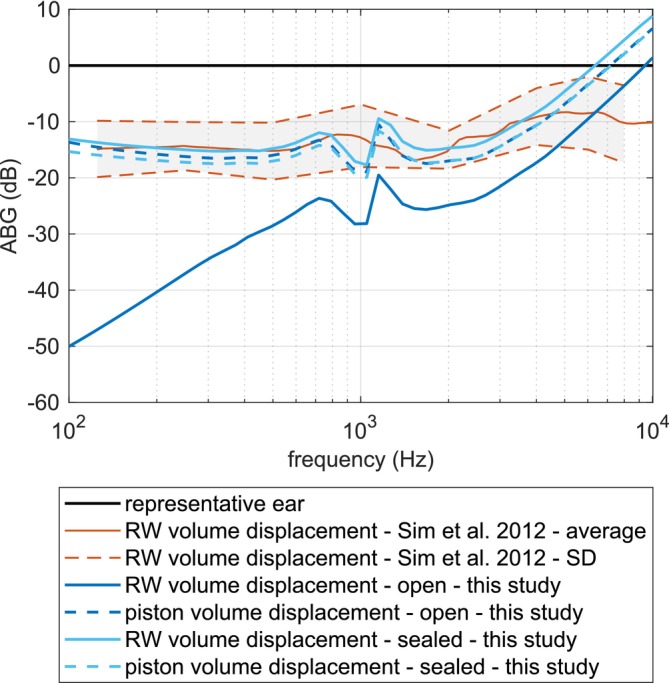
Different measures: volume displacement at RW and OW—simulation versus measurements.

Figure 19 shows how the simulated results for different boundary conditions at the hole in the SF—open and sealed—compare to the RW volume displacement measured on 15 temporal bones for normal and reconstructed ears in Sim et al. [2]. To simulate the sealed gap, the material properties of the connective tissue were assumed to be those of a tympanic membrane. According to Cheng [33], Rohani et al. [34], Zhang and Gan [35], and Luo et al. [36], the Young's modulus of the TM ranges from 0.4 to 58.9 MPa depending on prestress, frequency, and direction. The Young's modulus value in this study was assumed to be 3 MPa. In this case, there is little to no loss of pressure through the gap and the RW and piston volume displacement curves have roughly the same magnitudes which correspond well to the measurements from Sim et al. [2]. This means that, in cases where the gap is sealed, it would be enough to measure the displacement of the prosthesis itself in order to avoid the more difficult RW volume displacement measurements. This, however, will depend on the extent to which the gap is sealed.

In the case of an open gap, the RW volume displacement magnitudes are much lower compared to other measures, since there is a pressure loss through the open gap. This difference between the piston and RW volume displacements becomes smaller with the increase of the frequency. This can be explained by the closure of the gap at higher frequencies due to the increasing influence of the inertia of the fluid in the gap.

**FIGURE 20:**
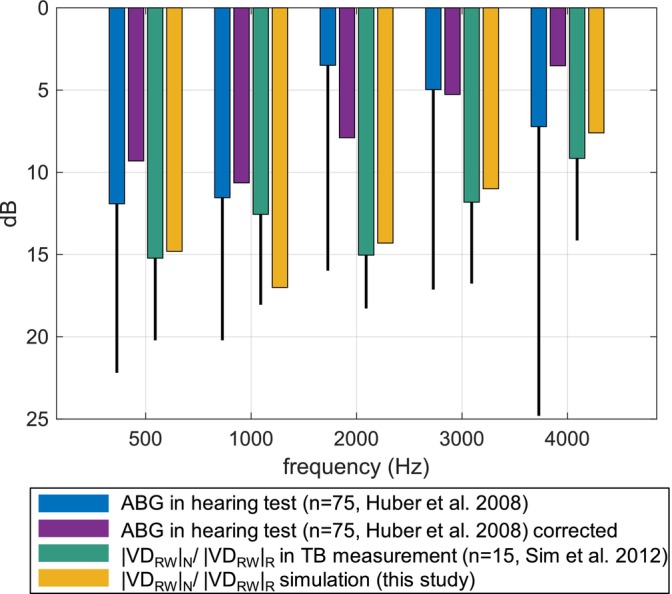
Air–bone gaps from hearing tests in patients after stapedotomy (*n* = 75) and magnitude ratios of volume displacements at the RW between normal (|VD_RW_|_N_) and reconstructed (|VD_RW_|_R_) ears in temporal bone measurements (*n* = 15) and simulation. Thin lines represent the standard deviations.

Figure 20 shows how the clinical data for the ABG after stapedotomy [2, 31] compared to the simulated magnitude ratio of the volume displacements at the RW between normal and reconstructed ears in the case of a sealed gap.

The expected postoperative ABGs in this study and Sim et al. [2] were calculated assuming that the postoperative BC hearing in patients is the same as BC hearing in normal ears. The simulated ABG fits well to the temporal bone measurements from Sim et al. [2]. The largest difference is 5 dB at 1 kHz. The ABG in Huber [31] and Sim et al. [2] was calculated using the pre‐stapedotomy BC gain. The average values of the ABG were corrected using the data for the post‐stapedotomy BC gain from Quaranta et al. [37] to be able to compare them to the data from Sim et al. [2], who also used post‐stapedotomy BC gain values to calculate the ABG, and to the simulated data from this study. As Quaranta et al. [37] only published the mean values, the standard deviation values are omitted.

Carhart [38] reported a depression of BC in patients with stapedial fixation, which is, on average, 5 dB at 0.5 kHz, 10 dB at 1 kHz, 15 dB at 2 kHz, and 5 dB at 4 kHz. Such frequency‐dependent loss of BC may be reversed by stapes surgeries. The improvement in hearing may be contributed to the restoration of the mobility of the middle‐ear ossicles, whose inertia is one of the five factors contributing to BC hearing according to Stenfelt [39]. Since the BC is not accounted for in the FE model, the effect of the recovery of the so‐called Carhart notch is not present in the simulated ABG. By correcting the average ABG from Huber [31] using postoperative BC from Quaranta et al. [37], the Carhart notch is no longer present in the ABG. The corrected data from Huber support the simulation results of this study quite well. When compared to the simulated results, the corrected ABG from Huber [31] has the same characteristic, but it is systematically smaller in magnitude, which could be explained by the different material and geometrical properties of the sealing membrane used in this study. In addition, a difference of 5 dB can be attributed to inaccuracies in audiometric measurements. The FE model of the middle and inner ear is suited to predict the general characteristic of postoperative ABG, assuming that BC hearing in reconstructed ears is the same as the BC hearing in normal non‐pathological ears.

## 5 Conclusions

A combined FE model of middle and inner ear was developed and validated using measurements from literature. The FE model can be used to investigate middle‐ear surgeries with regard to their effectiveness using several measures such as maximum BM displacement, intracochlear pressure, and volume displacement at the RW and OW. The FE model is suited to predict the ABG, however, it cannot predict the influence of BC changes like postoperative recovery of the Carhart effect in case of stapes prostheses.

## Ethics Statement

The authors have nothing to report.

## Consent

The authors have nothing to report.

## Conflicts of Interest

The authors declare no conflicts of interest.

## Supporting information


**Table S1.** Inner‐ear model material and damping properties.

## Data Availability

The data that support the findings of this study are available from the corresponding author upon reasonable request.
