# Supplementary material for: Coupled Finite Element Model of the Middle and Inner Ear as Virtual Test Environment for Stapes Surgery
Source: Int J Numer Method Biomed Eng. 2025 Feb 3;41(2):e70013. doi: 10.1002/cnm.70013 (PMC11790512; doi:10.1002/cnm.70013)
Supplement: Supplementary file 1 — Table S1. Inner‐ear model material and damping properties. [file CNM-41-e70013-s001.docx]

Table S1: Inner-ear model material and damping properties

| **Structure** | **Properties** |
| --- | --- |
| ***Stapes footplate (SF)*** |  |
| Density (kg/m^3^) | 2.30*10^3^ |
| Young’s modulus (N/m^2^) | 1.71*10^10^ |
| Damping | *α* = 0 s^−1^, *β* = 0*.*00005 s |
| ***Round window membrane (RW)*** |  |
| Density (kg/m^3^) | 1.20*10^3^ |
| Young’s modulus (Pa) | 3.50*10^5^ |
| Damping | *α* = 0s^−1^, *β* = 0.00005 s |
| ***Basilar membrane (BM)*** |  |
| Density (kg/m^3^) | 1.20*10^3^ |
| Young’s modulus (N/m^2^) | Equation (1) |
| Damping (beta) | Equation (2) |
| ***Perilymph fluid*** |  |
| Density (kg/m^3^) | 1.00*10^3^ |
| Bulk modulus (Pa) | 2.2*10^9^ |
